# The COVID-19 Lockdown and Mental Wellbeing of Females in China

**DOI:** 10.3390/ijerph19094960

**Published:** 2022-04-19

**Authors:** Chang-Lan Xia, An-Pin Wei, Yu-Ting Huang

**Affiliations:** 1 Faculty of Business, City University of Macau, Macau 999078, China; b13092300067@cityu.mo; 2 Department of Business Management, National Taichung University of Science and Technology, No. 129, Sec. 3, Sanmin Rd., North Dist., Taichung 404336, Taiwan; 3 Bachelor Program of International Management, National Yunlin University of Science and Technology, Yunlin 640301, Taiwan; ythuang@yuntech.edu.tw

**Keywords:** mental health, married females, lockdown, COVID-19

## Abstract

Most studies consider that COVID-19 lockdowns lead to mental health problems for females, while the effect of role change on female mental health has been overlooked. This study aimed to explore multiple facets of the risk of mental distress in a sample of Chinese married females aged 21–50 during the COVID-19 lockdowns. A cross-sectional study was carried out with 613 valid responses from married females in the Guangdong province. Our primary tool was a questionnaire using a Kessler-10 scale to detect the probability of mental distress based on the level of nervousness, tiredness, restlessness, and depression. Eighty-eight point three percent of married females possessed a high risk of psychological distress because they frequently felt tired out, hopeless, and restless. The evidence suggests that the lockdown has caused a conflict in the female role to maintain a balance between family and career. Increasing family care responsibilities are positively associated with nervousness, tiredness, and mental disorder. The heterogeneity of the social role in mental wellbeing is explored. Married females whose income was worse off during the lockdown are negatively associated with mental wellbeing. Married females who are employed are found to be less mentally healthy than the self-employed.

## 1. Introduction

Non-pharmaceutical interventions (NPI) were conducted by many counters to limit social contact and control the spread of the COVID-19 pandemic [1]. China chose to implement a lockdown policy, which is one of the most important NPI measures to avoid a massive spread of COVID-19, including limiting unnecessary social contact and travel, stay at home orders, closing school, work from home, learning online courses, and so on. Some research points to poor outcomes caused by the lockdown policy, such as increased unemployment and domestic violence [2]. A similar problem concerning married females during the lockdowns has been reported in a research paper; the work performance of married females became poorer than male colleagues when comparing publications by academic workers [3]. This has led to a heated debate: first, married females had to share more responsibilities of childcare because children had to stay at home; second, married females faced more family care because it was impossible to get home service from the market; last but not the least, the isolation changed the working and social environment [3].

In addition, substantial evidence has been found concerning mental health which was likely to be impacted during lockdown, including but not limited to anxiety, depression, sleeplessness, worry, and stress [4,5,6,7,8,9,10]. The psychological distress levels were promoted after a few months of lockdown in the USA, Germany, and the UK [11,12,13]. There exists a gender difference in the patterns of psychological distress and psychiatric disorder in studies before COVID-19 pandemic [14]. However, females are more vulnerable to mental health problems because the rates of common mental disorders in women are much higher than the rates of men [14]. According to World Health Organization, the occurrence of unipolar depression in women is twice as common as in men [15]. A recent study points out that women suffered more depression symptoms and disorders due to the lockdown in the Netherlands [16]. A cross-sectional study regarding health care workers during the COVID-19 pandemic in China was conducted. The result illustrates that the female gender tended to experience more severe depression, anxiety, and distress [17]. Bad mental conditions are associated with significant negative outcomes, such as relationship disruption, unstable employment, somatic illness, and suicide [1,18].

The structural gender differences induce gender differences in mental health during COVID-19 lockdown [17]. For example, the poor mental health of women may stem from discrimination and marginalization in the workplace [19,20,21]. Women are more likely to have a lower salary [21,22] because women are primarily defined as caregivers in society [23,24]. Women are less likely to be promoted to high-income subspecialties [22,25]. Women have to bear more family responsibilities compared to their male counterparts during the COVID-19 pandemic because of discrimination in duties [3]. In that way, social isolation, work disruption, financial worries, and health concerns due to the COVID-19 pandemic compound the stressors for women [21].

The main aim of this study is to explore the relationship between COVID-19 lockdowns and female mental wellbeing in China from the perspective of the social roles of females in China. China has promoted gender equality through laws, education, and government policies for decades. Females may be able to fill the labor shortage due to the aging population in China. In that way, this study hopes to assess the occurrence of gender role equalization in China during the lockdown period and how the changes in female roles affect mental wellbeing. The specific study process includes dividing the female social responsibilities into family care and work. A few factors, including family care time, job type, and annual income, during the lockdown period are investigated in this study.

The study makes several contributions to the research into Chinese female mental wellbeing. First, the family role is also investigated instead of focusing on only the career aspect. New evidence concerning the mental wellbeing of Chinese females using data from a lockdown survey is provided in this research. The survey covered married females of the Guangdong province, where both males and females tend to share fair social roles in a society with fast social and economic development. Married females who had a full-time jobs were selected because the study focuses on females who have to balance both work and family roles during the lockdown period. Second, a theoretical and empirical foundation is established to link work motives to female happiness. The underlying mechanism of female mental wellbeing could be revealed on the basis of exploring job role measurements. New exploration concerning female wellbeing and life role is complemented both from a theoretical and empirical perspective.

## 2. Literature Review

Gender ideology can help explain structural gender differences because gender ideology sets stereotypes for different genders [26,27,28]. The gender stereotype is used to define the life roles and psychology men and women abide by, including but not limited to different social norms, behavior patterns, and social functions [26,27]. There are mainly two gender ideologies in the present society. Traditional gender ideology defines gender stereotypes based on biological and social characteristics [27]. In traditional gender ideology, males have physical advantages over females and undertake responsibilities of clan inheritance and household management; on the other hand, females conduct childbearing responsibilities and rely on husbands and sons as wives and mothers [29]. There exists a significant gap between male and female roles. Specifically, females aim to bear children and take care of families because they are limited to family function compared to male partners [27,28]. It is called ‘male takes care of outside; female takes care of inside’ in Chinese sayings.

However, females have flocked into the labor force market after World War II, and the pattern of “only males provide bread” has been changing in society [29]. Gender roles have evolved with social functions in modern society, and gender discrepancy based on social functions is minute [27], because social functions of genders are created by human interaction instead of natural processes. Put another way, humans construct different gender roles for society [27]. A few scholars have suggested an ideology of “undoing gender” and “redoing gender” to resist traditional gender ideology [27,30]. The final goal of resisting traditional gender ideology will be the gender egalitarianism ideology, by which individual assessment will not be affected because of gender factors [27,31]. The gender role equalization ideology considers that both females and males have the same significance in different functions of society [27]. Thus, the psychology and behaviors of humans will be different under different gender ideologies.

By now, a few researchers have developed some dimensions to measure the equality of gender roles. The Sex Role Egalitarianism Scale suggests females should share equalized responsibilities of the Marital Role, Parental Role, Employment Role, Social–Interpersonal–Heterosexual Role, and Education Role with males [31]. Another approach is investigating four dimensions of gender equality, including the possibility to maintain a family, the capabilities to make choices, the resources to hire agents, and to share equal household work [32]. Both approaches show that responsibilities and behaviors of both genders converge in multiple areas during the process of gender role transition. In traditional gender role ideology, men are dominators and women are subordinates because women only undertake housework and family care, and men receive education and employment opportunities; in the equalized gender role ideology, women receive education and enter the labor market, while men share family care and housework [26,27,28,29,30]. It is noted that there is a significant difference in mental wellbeing due to the different individual psychology and behavior under different gender ideologies. For example, the mental health of females is significantly associated with their social status [14]. Females suffered a much higher probability of depression and anxiety than males because of unfair family and employment roles [33,34,35].

According to stress theory, psychological stress refers to a relationship with the environment; that is to say when a shock or a response happens to a person, negative psychology and effects are aroused, such as anxiety and depression [36]. If the negative emotions cannot be regulated actively, the individuals will remain in a state of stress for a long time, which will damage the physical and mental wellbeing and lead to serious mental problems such as posttraumatic stress disorder (PTSD) [37]. During the COVID-19 lockdown from the Lunar New Year 2020 to June 2020 in Guangdong in China, a mass quarantine policy was implemented. The citizens were not allowed to leave home unless for necessities, such as health problems, daily basic necessities, and work that could not be finished at home. The COVID-19 epidemic posed threats and concerns, which led to increased family care [3]. Females had to balance employment and family roles at home because of the COVID-19 lockdown. It is possible that the negative effects were aroused during the lockdown period [17,18,19,20,21]. In order to find out whether the COVID-19 lockdown negatively influenced depression and anxiety through changing family responsibilities, we propose to following hypotheses:

**Hypothesis** **1** **(H1).** *Females have more risk of mental disorders with more family care responsibilities.*

The career role is another potential factor that is significantly associated with mental wellbeing [3,38,39]. The Job Demand Control Support (JDCS) model establishes a two-dimension framework; on the one hand, job demands involve job requirements; on the other hand, job control means the ability to finish the job according to individual willingness [40,41,42]. Low mental wellbeing of workers will occur with the highest job demands and lowest job control [40,42]. That is to say, higher demands and lower control are significantly associated with anxiety [40,41,42]. Job control can be measured by authority and responsibilities [40]. It also indicates that the power of individual control has links to the degree of participation in organizational decision-making [42]. The self-determination theory also agrees that autonomy is one of the most important basic psychological needs for self-achievement [40,42,43]. When individuals have a higher level of control, their abilities, influence, and potential are realized more often [40,42,43]. The self-employed have been found to have a higher level of control than others, and hence their mental wellbeing is promoted by fulfilling the process of running a business [42,44].

Females have pursued the same employment opportunities and advancement under the gender egalitarianism ideology [3,27,31,32]. Career development has become one of the most significant life goals for women today [26,27,28,29,30], especially in an economically developed area such as the Guangdong province in China. However, the COVID-19 lockdown caused a shock to the working environment for females [3]. It was harder to control the job owing to the increasing family role and decreasing effort of female workers during the COVID-19 lockdown [3]. Compared to the self-employed, employees are more likely to lose control over their jobs, which could increase the possibility of mental disorders. Therefore, we proposed the following hypotheses:

**Hypothesis** **2** **(H2).** *Employed females have more risk of mental disorders than the self-employed.*

## 3. Materials and Methods

### 3.1. Data Sources and Sample Composition

This study was a cross-sectional analysis. After the lockdown of Wuhan in 2020, the COVID-19 pandemic spread around the country. Most provinces and cities undertook lockdown policies from the Chinese New Year. The lockdown policy was executed until the end of June in the Guangdong province. The survey was undertaken at the beginning of May, when the lockdown had been in place for a period of time.

The study mainly focuses on the relationship between responsibility change and the mental health of the females during the COVID-19 lockdown. To explore the impact of the lockdown on both the work and family care roles of females, the women who were both working and married were selected. Because Guangdong province is one of the most developed regions where women have equal opportunities to receive education and enter the labor market, we decided to undertake a survey of working married women in the Guangdong province. As the age of female retirement in China is 50, females under the age of 50 were selected for the survey. We recruited 643 women primarily living and working in Zhuhai, Shenzhen, Guangzhou, Dongguan, Jieyang, and Maoming of the Guangdong province by snowballing. Six hundred and forty-three participants attended the test voluntarily; however, only 613 participants completed the assessment with valid answers. The valid response rate reached 95%.

### 3.2. Variables and Definitions

#### 3.2.1. Dependent Variables

The main dependent variable used to assess the mental wellbeing of married females was the Kessler-10 scale, which contains different items of anxiety, depression, and psychological fatigue, and is a short measure of psychological distress widely used in the US National Health Interview Survey [45,46]. The Kessler-10 scale has great reliability and validity in China [47], and Cronbach’s Alpha reached 0.846 in the article.

The scale consisted of 10 question items, including “During the lockdown period, how often did you feel tired out for no good reason?”, “During the lockdown period, how often did you feel nervous?”, “During the lockdown period, how often did you feel too nervous to calm down yourself?”, “During the lockdown period, how often did you feel desperate ?”, “During the lockdown period, how often did you feel restless?”, “During the lockdown period, how often did you feel too restless to stay still?”, “During the lockdown period, how often did you feel depressed?”, “During the lockdown period, how often did you feel that it is hard to finish the thing?”, “During the lockdown period, how often did you feel so sad that nothing could cheer you up?”, and “During the lockdown period, how often did you feel worthless?” Each item is scored from 1 = “none of the time” to 5 = “all of the time”.

The general mental health could be analyzed by summing the scores of the 10 items, yielding a minimum score of 10 and a maximum score of 50 [45,46]. The level of psychological distress problems could be categorized into 3 types according to total scores: general mental wellbeing is likely to be well if the total score is equal or below 19, medium risk of mental distress is suggested when the total score is between 20 and 29, and high risk of psychological distress exists if the total score is above 29 [46].

#### 3.2.2. Independent Variables

As responsibility characteristics influence mental health, a few significant independent variables were chosen. First, the researcher checked whether married females had higher family care responsibilities. Hence, the independent variables included female family care hours per day, the increasement of female family care time, and husbands’ family care hours per day during lockdown. Both female family care hours and husbands’ family care hours per day during the lockdown period were measured as 1 = “1 h or below”, 2 = “2–3 h”, 3 = “4–5 h”, 4 = “6–7 h”, and 5 = “7 h above”. During the lockdown, the increment of female family care time was classified as 0 = “without more family care” and 1 = “with more family care”. Moreover, the child factor (0 = “without child” and 1 = “with child”) was considered as a potential factor that is correlated with family care responsibility.

During lockdown, the work responsibilities were obtained by variables of job characteristics, including female job type, female annual income, decrease in female income, and partners’ annual income. The job type variable was classified as 0 = “self-employed” and 1 = “employee”. Both female annual income and partners’ annual income were classified as 1 = “RMB (Renminbi) 50,000 or below”, 2 = “RMB 50,001–100,000”, 3 = “RMB 100,001–150,000”, 4 = “RMB 150,001–200,000”, 5 = “RMB 200,000 or above.” The decrease in female annual income was measured by “Has your income decreased during the lockdown period?” (0 = “without less income;” 1 = “with less income”).

Several variables, which might be correlated with mental wellbeing commonly in the research, were controlled to reduce potential confounding effects. The personal characteristics were measured by a region of registration (“hukou”) variable (0 = rural, 1 = urban) [42] of married females, the age group variables for both females and partners (1 = 21–30, 2 = 31–40, 3 = 41–50, 4 = above 50) [42], and education (1 = high school or below, 2 = college, 3 = bachelors, 4 = masters, 5 = doctor) variable for both wives and husbands [48].

### 3.3. Statistical Analysis

All questionnaire data were processed and analyzed with the computer software STATA 14 (Stata Corp., College Station, TX, USA). Descriptive statistics were conducted for the data. Considering most of the variables were categorical variables with clear orderings, the method for multivariable analysis was ordinal logistic regression models. Ordinal logistic regression is a statistical analysis method that can be used to model the relationship between an ordinal response variable and one or more explanatory variables. The odds ratio of logistic regression is a significant way to compare the possibility of psychological distress with different dependent variables.

## 4. Results

Table 1 shows the description of participants in the total sample, including the frequency and distribution of different variables and characteristics. Among the 613 married females mainly living and working in the Guangdong province, 445 were self-employed and 168 were employees. Seventy point one percent were registered with rural “hukou”, 62.6% were living with children, 75.7% were between age 20 and 40, 50.4% were working 3 h or below in family care per day during the lockdown, and only 36.7% felt that family care time went up during the lockdown. Most females earned an annual income “RMB 50,001–100,000”, and the ratio of females with “RMB 50,001–100,000” reached about 30.8%. Eighty-five percent of married females reflected that income declined during the COVID-19 lockdown. Approximately 51.2% of females had completed college or lower. Information concerning their husbands: 51.8% finished college or lower, and 76.1% were between the age of 20 to 40. Most husbands undertook 4–5 h of family care, which reached 30.7%. Most husbands earned an annual income of “RMB 50,001–100,000”. The psychological distress among married females was disturbing. The majority were at high risk of mental distress (88.9%), and only 0.3% of married females felt very well during the lockdown. Over 70% of females felt “Difficult to calm down”, “Tired out”, “Hopeless”, “Depressed”, “Difficult to stay still”, “Hard to finish anything”, “Restless”, “Difficult to cheer up”, and “Worthless” most of the time or all the time. It appears that most married females needed clinical psychology therapy after a few months of lockdown.

To further analyze the relationship between negative effects and females’ responsibilities during lockdown, the ordinal logistic regression results of the general mental health valuation and each item of the scale will be presented. Table 2 displays the odds ratios (O.R.) and corresponding 95% confidence interval (C.I.) of “nervous”, “difficult to calm down”, and “tired out” by different resident characteristics in the total sample. Females who had more family care time during the lockdown felt nervous more frequently (O.R. = 4.639; 95% C.I. = 3.307–6.509). Females whose income dropped during the lockdown were more nervous than females whose income did not drop (O.R. = 1.78; 95% C.I. = 1.18–2.69). The job role of married females was significantly associated with the negative effect “difficult to calm down”. Employees who had lower control over their job were more difficult to calm down than self-employed females (O.R. = 2.001; 95% C.I. = 1.894–2.09), and decreasing income was more likely to increase the difficulty to calm down (O.R. = 1.793; 95% C.I. = 1.163–2.766). On the contrary, the family role of married females did not show a negative association with difficulty to calm down. The longer the hours females undertook family care during the lockdown, the less was the difficulty to calm down (O.R. = 0.849; 95% C.I. = 0.752–0.958). Both family role and job type were significantly related to a “tired out” feeling. Employed females were more tired than the self-employed during the lockdown (O.R. = 1.54; 95% C.I. = 1.085–2.185). Females with more family care time were more tired than those without during the lockdown (O.R. = 1.819; 95% C.I. = 1.317–2.512). However, women who had children to take care of were less likely to feel tired out than those who did not (O.R. = 0.71; 95% C.I. = 0.68–0.758).

The dependent variables “nervous”, “difficult to calm down”, and “tired out” were replaced with negative influence “hopeless”, “depressed”, and “difficult to sit still” in the Table A1. The result showed that job type had a significant influence on female’s negative feeling of hopelessness, depression, and hard to sit still during lockdown. Employees were more likely to feel hopeless (O.R. = 1.581; C.I. = 1.119–2.235), depressed (O.R. = 2.709; 95% C.I. = 1.887–3.889), and hard to sit still (O.R. = 1.419; C.I. = 1.119–2.235) than the self-employed. The annual income of females was positively associated with the odds of feeling hopeless during the lockdown (O.R. = 1.169; 95% C.I. = 1.038–1.316). Females who took on more responsibilities for family care felt more depressed than those who did not (O.R. = 2.447; 95% C.I. = 1.759–3.404).

The dependent variables were replaced by the negative influence of “hard to finish things”, “restless”, and “difficult to cheer up” in Table A2. Similarly, job type is significantly associated with the odds of negative effects aforementioned. Employed females were more likely to feel difficult to finish the work (O.R. = 1.634; 95% C.I. = 1.154–2.314) and restless (O.R. = 1.795; 95% C.I. = 1.259–2.559). The annual income of females was positively associated with odds of restlessness (O.R. = 1.167; 95% C.I. = 1.035–1.31) during the lockdown. The education of females was significantly related to feeling difficult to cheer up. The higher the level of education females reached, the more difficult to cheer up during lockdown (O.R. = 1.174; 95% C.I. = 1.04–1.325).

Table A3 shows the odds ratios of worthless and general mental distress by different resident characteristics in the sample. It is indicated that self-employed females were less likely to feel worthless than employed (O.R = 1.574; 95% CI = 1.108–2.235). The higher the female’s annual income, the more frequently they were to feel worthless (O.R. = 1.185; 95% C.I. = 1.051–1.335). The job characteristics and family care had a significant influence on the general mental distress of married females. Increasing house care time and annual income were more likely to increase the odds of mental problems for married females (O.R. = 2.023; 95% C.I. = 1.484–2.757). Employed females were 1.5 times more likely than the self-employed to have mental distress concerns (O.R. = 2.573; 95% C.I. = 1.835–3.608) during the lockdown. On the one hand, the more annual income females had, the more risk of mental disorders females had (O.R. = 1.149; 95% C.I. = 1.029–1.283). On the other hand, women with falling income during the lockdown probably experienced more risks of mental problems than those without falling income (O.R. = 1.601; 95% C.I. = 1.074–2.387). From the evaluation of Table 2 to Table A3, H1 and H2 were generally supported. The mental distress risk of females resulted from an increasing family care role and employed job category.

## 5. Discussion

Compared to males, females are anticipated to suffer from harsh working environments and complicated mental health problems around the world because of gender role differences [1,16,17]. In the process of gender role transition, females substantially tend to develop their career paths and share family care roles with partners. However, the COVID-19 lockdown has changed the existing social and working environment; females were pushed to share more family care responsibilities and deal with jobs at home. Most previous research implies the wellbeing of females will suffer from unfair family care roles [16,17,18] and lower control over work [42,43]. The employees who could not make their own business decisions had fewer opportunities to dominate, compared with the enterprisers who are the decision-makers. Based on the related theory, the study drew the hypotheses on the relationship between different life roles and mental wellbeing. It turned out that the impact on mental wellbeing varied according to different family and job responsibilities stemming from different types of jobs.

Using a cross-sectional survey in the Guangdong province of China, the outcomes of this study provided new evidence regarding female mental wellbeing under the COVID-19 lockdown policy. First, the risk of mental distress was fairly high among the married females who reported increasing family care time during the lockdown. Second, females who had more family care time more frequently felt nervous, tired out, and depressed than those who did not during lockdown. Third, the family care hours per day were positively associated with the difficulty of calming down. The more hours per day women undertook during the lockdown, the less likely women were able to calm down the nervous feeling. Moreover, although the average family care hour of husbands was almost equal to the average family care hour of females during lockdown, the family care hours per day of husbands had no significant influence on females’ mental wellbeing factors. It is possible that although males are required to share part of home caring responsibilities during the lockdown, it may not be quality care time and females have increase time to address home affairs. Children were not a significant stressor in the survey.

The characteristic of the job was taken into account to explore the reasons why females were exposed to a high risk of mental disorder during the lockdown. Self-employed are always considered to have higher control over their job than employees [48,49]. Over half of the married females chose a self-employed job in the survey, possibly to balance work and family. Employees were more likely to need clinical psychological therapy than the self-employed. Female employees were more frequently felt difficult to calm down, tired out, hopeless, depressed, difficult to sit still, restless, worthless, and so on. The average annual income of females was a bit higher than the average income of husbands in the survey. The more annual income females had, the higher the probability of mental distress, such as feelings of hopelessness, restlessness, and worthlessness. Compared to females whose income was not affected by the pandemic, females whose income was reduced had serious mental distress, such as they feel too nervous to calm down most of the time. It is possible that women take significant roles in providing financial support for the family. Decreasing income probably leads to a decline in living standards, especially for females with a higher level of income.

Noteworthy, the mental wellbeing of married females was worse in the higher levels of lockdown restrictions. This finding indicated the concordant results from recent studies, which conclude the overall influence on the mental wellbeing of humans because of COVID-19 limitations [1,50]. China is a typical country that is experiencing a gender revolution. Females started to share equal roles with males, especially in developed regions such as Beijing and Shanghai. When the woman plays a significant role in the financial support of the family, the redistribution of effort between family and work will have a negative influence on psychological distress during the lockdown. It emphasizes the necessity of cautious assessment of the relative harms and continuing analysis for cost–benefit when the lockdown is taken into account [1,51]. Furthermore, NPIs, such as lockdown, may unleash adverse sequelae on health and life [1,52,53]. The demand for mental health support shows an increasing trend in aspects of both community and society [1]. After the pandemic for females whose social roles have been disproportionately affected, more planning and investment in mental health support should be considered. It is suggested that reinforcing mental health systems and improving investment for health workers will be beneficial to ease poor outcomes caused by the epidemic, as well as enhance the ability to recover mental health systems in the future [1,54].

## 6. Limitations and Contribution

The shortcomings of the data and sample method lead to the limitations of this study. A cross-sectional study of mental wellbeing, which refers to a self-reported survey, was conducted. The data were limited because it only came from Guangdong which is one of the most developed provinces in China. The research is limited in time, and longitudinal data, which will provide more predictive utility of mental health outcomes, will be needed. Second, the family and career roles we constructed were based on past studies, which may have lacked the convincing power of measures under gender egalitarianism ideology in the Guangdong province. Another limitation is the measures, given the difficulties investigating married women through the lockdowns. Moreover, qualitative studies can be implemented to explore more details of females’ experiences through COVID-19 lockdowns. Third, when examining the difference in various mental wellbeing, individual heterogeneity and selection were not completely controlled. For instance, family support and social support, which vary among the females, could mitigate the negative effects of lockdown. The contrast of individual characteristics may also affect the distribution of psychological states, such as neuroticism or extraversion. Finally, the statistical analysis method is limited to a single regression methodology. Other analysis methodologies should be used to increase the accuracy of the moderating effect mechanism.

However, the study contributes to the research into female mental health in a few ways. First, we examined the impact of the pandemic on mental health by developing a new framework that draws attention to gender role. While other studies found that females were unhappy when females take more family responsibilities and lower career roles, this research found that the equalized responsibilities of both family and work could induce the risk of mental distress as well. Another strength of the present study is providing important information on mental welling during the COVID-19 pandemic. The study captures the multi-important indices of mental health for females, including nervousness, depression, worthless, restlessness, and tiredness. Additionally, this study enriches the insufficient research on the severe impact on females’ daily lives due to COVID-19 lockdowns. The lockdown had adverse effects on both the job and family roles of females, especially for the employed. Women who had low control over the job and more family responsibilities were most likely to suffer mental distress.

## 7. Conclusions

In summary, we report a study examining the mental wellbeing for females during the lockdown. The evidence suggested that lockdowns are likely to cause psychopathology by influencing the family and job roles. During the lockdown, less income and more family care will be the main factors causing negative mental wellbeing. Women who are employed are more difficult to calm down and complete the task. The probability of clinical need will be accumulated because of negative emotions of nervousness, tiredness, restlessness, and so on. We appeal to the policymakers to think twice when considering lockdown strategies in the future. We also hope that social support, as well as medical interventions, will be provided to mitigate the possible damage to health and wellbeing. Humans should be prepared for potential clinical needs because the pandemic has created a cost to social life.

## Figures and Tables

**Table 1:** Frequency and distribution of the total sample.

Characteristics		*n*	Ratio (%)	Mean (S.D.)
age	21~30 years	240	39.2	1.85 (0.784)
	31~40 years	224	36.5	
	41~50 years	149	24.3	
Education	High school or below	156	25.4	2.56 (1.257)
	college	158	25.8	
	bachelor	147	24	
	master	102	16.6	
	doctor	50	8.2	
Region of registration	Rural	430	70.1	0.30 (0.458)
	Urban	183	29.9	
Job type	Self-employed	445	72.6	0.27 (0.466)
	employee	168	27.4	
Family care hours per day	≤1 h	135	22	2.61 (1.245)
	~3 h	174	28.4	
	~5 h	157	25.6	
	~7 h	87	14.2	
	>7 h	60	9.8	
More family care hours	without	388	63.3	0.37 (0.482)
	with	225	36.7	
child	without	299	37.4	0.63 (0.484)
	with	384	62.6	
Less income	without	92	15	0.85 (0.357)
	with	521	85	
Annual income	≤50,000 RMB	134	21.9	2.61 (1.260)
	~100,000 RMB	189	30.8	
	~150,000 RMB	137	22.3	
	~200,000 RMB	90	14.7	
	>200,000 RMB	63	10.3	
Husband education	High school or below	126	20.6	2.59 (1.219)
	college	191	31.2	
	bachelor	163	26.6	
	master	72	11.7	
	doctor	61	10	
Husband age	21~30 years	243	39.6	1.97 (1.011)
	31~40 years	224	36.5	
	41~50 years	67	10.9	
	50 years above	79	12.9	
Husband annual income	≤50,000 RMB	143	23.3	2.55 (1.239)
	~100,000 RMB	180	29.4	
	~150,000 RMB	155	25.3	
	~200,000 RMB	77	12.6	
	>200,000 RMB	58	9.5	
Husband family care hour per day	≤1 h	107	17.5	2.78 (1.238)
	~3 h	157	25.6	
	~5 h	188	30.7	
	~7 h	87	14.2	
	>7 h	74	12.1	
Nervous	None of the time	3	0.5	3.64 (0.936)
	A little of the time	60	9.8	
	Some of the time	221	36.1	
	Most of the time	201	32.8	
	All of the time	128	20.9	
Difficult to calm out	None of the time	7	1.1	3.94 (0.884)
	A little of the time	29	4.7	
	Some of the time	131	21.4	
	Most of the time	275	44.9	
	All of the time	171	27.9	
Tired out	None of the time	1	0.2	4.04 (0.849)
	A little of the time	21	3.4	
	Some of the time	140	22.8	
	Most of the time	242	39.5	
	All of the time	209	34.1	
Hopeless	None of the time	7	1.1	4.01 (0.934)
	A little of the time	43	7	
	Some of the time	94	15.3	
	Most of the time	264	43.1	
	All of the time	205	33.4	
Depressed	None of the time	3	0.5	3.76 (0.837)
	A little of the time	26	4.2	
	Some of the time	210	34.3	
	Most of the time	251	40.9	
	All of the time	123	20.1	
Difficult to stay still	None of the time	5	0.8	4.00 (0.929)
	A little of the time	43	7	
	Some of the time	106	17.3	
	Most of the time	254	41.4	
	All of the time	205	33.4	
Hard to finish the thing	None of the time	16	2.6	4.02 (0.970)
	A little of the time	21	3.4	
	Some of the time	122	19.9	
	Most of the time	228	37.2	
	All of the time	226	36.9	
Restless	None of the time	8	1.3	4.05 (0.875)
	A little of the time	27	4.4	
	Some of the time	92	15	
	Most of the time	288	47	
	All of the time	198	32.3	4.06 (0.852)
Difficult to cheer up	None of the time	6	1	
	A little of the time	14	2.3	
	Some of the time	127	20.7	
	Most of the time	257	41.9	
	All of the time	209	34.1	
Worthless	None of the time	23	3.8	3.98 (1.011)
	A little of the time	28	4.6	
	Some of the time	96	15.7	
	Most of the time	255	41.6	
	All of the time	211	34.4	
General mental wellbeing	Well (1~20)	2	0.3	
	Moderate risk of mental distress (21~30)	66	10.8	39.49 (5.89)
	High risk of mental distress (30 above)	545	88.9	

**Table 2:** Ordinal logistic regression of negative affects partially with different factors in the total sample.

	Nervous						Difficult to Calm Out						Tired Out					
Characteristics	Odd Ratio	S.E.	*t*-Value	*p*-Value	[95% Conf. Interval]		Odd Ratio	S.E.	*t*-Value	*p*-Value	[95% Conf. Interval]		Odd Ratio	S.E.	*t*-Value	*p*-Value	[95% Conf. Interval]	
FCHPD	1.027	0.062	0.44	0.659	0.912	1.157	0.849	0.052	−2.66	0.008	0.752	0.958	0.986	0.059	−0.23	0.820	0.877	1.110
More family care	4.639	0.801	8.88	0.000	3.307	6.509	1.183	0.195	1.02	0.307	0.857	1.633	1.819	0.300	3.63	0.000	1.317	2.512
Job type	1.379	0.247	1.80	0.072	0.976	1.958	2.001	0.364	3.81	0.000	1.401	2.859	1.540	0.275	2.41	0.016	1.085	2.185
Annual income	1.103	0.066	1.64	0.13	0.981	1.241	1.033	0.061	0.55	0.585	0.919	1.160	1.051	0.063	0.83	0.404	0.935	1.182
Less income	1.780	0.374	2.75	0.006	1.180	2.69	1.793	0.397	2.64	0.008	1.163	2.766	1.397	0.307	1.52	0.129	0.908	2.149
Child	0.903	0.14	−0.65	0.514	0.666	1.225	1.014	0.150	0.09	0.930	0.747	1.377	0.717	0.002	−11.88	0.000	0.680	0.758
Age	0.996	0.096	−0.04	0.968	0.824	1.203	0.989	0.096	−0.11	0.910	0.818	1.195	0.908	0.087	−1.00	0.317	0.751	1.097
Region	1.111	0.183	0.64	0.523	0.804	1.535	0.948	0.156	−0.33	0.743	0.687	1.308	1.299	0.215	1.58	0.114	0.939	1.798
Education	0.973	0.059	−0.45	0.652	0.863	1.096	0.952	0.058	−0.81	0.417	0.845	1.072	1.087	0.065	1.39	0.165	0.966	1.223
Hus-FCHPD	1.094	0.068	1.44	0.150	0.968	1.096	0.067	1.490	0.136	0.971	1.237	1.115	1.015	0.063	0.23	0.815	0.899	1.145
Hus-education	1.006	0.062	0.10	0.923	0.891	1.135	1.060	0.067	0.93	0.354	0.937	1.199	0.986	0.062	−0.23	0.817	0.872	1.114
Hus-age	0.976	0.074	−0.32	0.752	0.842	1.132	1.015	0.077	0.20	0.841	0.874	1.178	0.910	0.068	−1.26	0.208	0.785	1.054
Hus-income	1.044	0.064	0.70	0.483	0.926	1.176	0.944	0.058	−0.93	0.350	0.838	1.065	1.018	0.063	0.29	0.775	0.902	1.149
Less hus-income	1.251	0.252	1.11	0.266	0.843	1.858	0.811	0.165	−1.03	0.303	0.544	1.209	0.799	0.162	−1.10	0.269	0.536	1.190

Notes: FCHPD, family care hours per day during lockdown; Hus-FCHPD, husbands’ family care hours per day during lockdown; working hours per week; Hus-education, education of husband; Hus-age, age of husband; Hus-income, annual income of husband; Less hus-income, whether the annual income of husband decreased during the lockdown.

## Appendix A

**Table A1:** Ordinal logistic regression of “hopeless”, “depressed”, and “difficult to sit still” partially with different factors in the total sample.

	Hopeless						Depressed						Difficult to Sit Still					
Characteristics	Odds Ratio	S.E.	*t*-Value	*p*-Value	[95% Conf. Interval]		Odds Ratio	S.E.	*t*-Value	*p*-Value	[95% Conf. Interval]		Odds Ratio	S.E.	*t*-Value	*p*-Value	[95% Conf. Interval]	
FCHPD	0.943	0.058	−0.96	0.338	0.837	1.063	1.038	0.065	0.60	0.547	0.919	1.173	1.084	0.066	1.34	0.181	0.963	1.221
More family care	1.015	0.164	0.09	0.925	0.740	1.392	2.447	0.412	5.31	0.000	1.759	3.404	1.024	0.167	0.15	0.883	0.745	1.409
Job type	1.581	0.279	2.59	0.009	1.119	2.235	2.709	0.500	5.40	0.000	1.887	3.889	1.419	0.252	1.97	0.049	1.002	2.010
Annual income	1.169	0.071	2.57	0.010	1.038	1.316	1.023	0.062	0.38	0.704	0.909	1.152	1.073	0.064	1.17	0.241	0.954	1.207
Less income	1.030	0.222	0.14	0.892	0.675	1.571	0.789	0.172	−1.09	0.276	0.515	1.209	0.942	0.203	−0.28	0.782	0.618	1.437
Child	0.994	0.156	−0.04	0.971	0.731	1.352	1.112	0.177	0.67	0.504	0.814	1.519	0.922	0.145	−0.51	0.607	0.678	1.256
Age	0.836	0.087	−1.87	0.061	0.693	1.009	0.918	0.089	−0.88	0.378	0.758	1.111	1.032	0.099	0.33	0.744	0.855	1.245
Region	1.060	0.174	0.36	0.721	0.768	1.463	1.216	0.205	1.16	0.247	0.747	0.873	1.692	0.182	0.62	0.535	0.803	1.527
Education	1.033	0.063	0.54	0.590	0.918	1.163	0.061	0.065	0.97	0.333	0.941	1.195	1.099	0.066	1.58	0.113	0.978	1.236
Hus-FCHPD	1.036	0.065	0.57	0.571	0.917	1.171	0.921	0.058	−1.31	0.189	0.814	1.041	0.979	0.061	−0.33	0.738	0.867	1.106
Hus-education	1.026	0.064	0.40	0.686	0.908	1.159	1.115	0.070	1.74	0.082	0.986	1.261	0.936	0.057	−1.08	0.281	0.831	1.055
Hus-age	0.884	0.068	−1.60	0.109	0.761	1.028	1.039	0.079	0.50	0.614	0.896	1.205	0.918	0.069	−1.14	0.255	0.792	1.064
Hus-income	1.039	0.064	0.61	0.542	0.920	1.173	0.914	0.057	−1.45	0.148	0.810	1.032	1.027	0.062	0.45	0.655	0.913	1.156
Less hus-income	0.919	0.188	−0.42	0.677	0.615	1.371	1.121	0.232	0.55	0.580	0.748	1.681	1.045	0.217	0.21	0.832	0.695	1.571

Notes: FCHPD, family care hours per day during lockdown; working hours per week; Hus-FCHPD, husbands’ family care hours per day during the lockdown; Hus-education, education of husband; Hus-age, age of husband; Hus-income, annual income of husband; Less hus-income, whether the annual income of husband decreased during the lockdown.

**Table A2:** Ordinal logistic regression of “hard to finish the thing”, “restless”, “difficult to cheer up” partially with different factors in the total sample.

	Hard to Finish the Thing						Restless						Difficult to Cheer Up					
Characteristics	Odds Ratio	S.E.	*t*-Value	*p*-Value	[95% Conf. Interval]		Odds Ratio	S.E.	*t*-Value	*p*-Value	[95% Conf. Interval]		Odd Ratio	S.E.	*t*-Value	*p*-Value	[95% Conf. Interval]	
FCHPD	0.938	0.057	−1.05	0.293	0.834	1.056	1.016	0.063	0.26	0.796	0.900	1.147	0.926	0.057	−1.25	0.212	0.820	1.045
More family care	0.797	0.128	−1.41	0.158	0.582	1.092	1.087	0.181	0.50	0.617	0.784	1.506	0.831	0.137	−1.12	0.261	0.602	1.148
Job type	1.634	0.290	2.76	0.006	1.154	2.314	1.795	0.325	3.23	0.001	1.259	2.559	1.196	0.212	1.01	0.314	0.845	1.693
Annual income	1.112	0.066	1.78	0.076	0.989	1.249	1.167	0.072	2.52	0.012	1.035	1.31	1.097	0.066	1.54	0.123	0.975	1.235
Less income	1.230	0.258	0.98	0.325	0.815	1.855	1.231	0.258	0.99	0.322	0.816	1.856	1.467	0.315	1.78	0.074	0.963	2.233
Child	0.935	0.146	−0.43	0.669	0.689	1.270	0.802	0.127	−1.39	0.163	0.587	1.094	1.006	0.157	0.04	0.967	0.741	1.367
Age	1.163	0.111	1.59	0.113	0.965	1.402	1.027	0.100	0.28	0.783	0.848	1.244	0.915	0.087	−0.93	0.352	0.759	1.103
Region	1.261	0.208	1.41	0.159	0.913	1.741	0.982	0.165	−0.11	0.915	0.707	1.365	0.857	0.140	−0.95	0.344	0.621	1.181
Education	1.018	0.061	0.31	0.760	0.906	1.145	1.009	0.062	0.14	0.887	0.894	1.138	1.174	0.073	2.59	0.010	1.040	1.325
Hus-FCHPD	0.945	0.058	−0.92	0.359	0.837	1.066	0.946	0.059	−0.88	0.377	0.836	1.07	0.886	0.056	−1.92	0.054	0.784	1.002
Hus-education	1.054	0.064	0.86	0.390	0.935	1.187	1.105	0.070	1.57	0.116	0.976	1.251	0.981	0.061	−0.31	0.753	0.868	1.108
Hus-age	0.931	0.070	−0.95	0.343	0.802	1.080	0.932	0.072	−0.91	0.364	0.801	1.085	0.923	0.070	−1.06	0.289	0.796	1.07
Hus-income	1.028	0.063	0.45	0.653	0.912	1.159	0.933	0.058	−1.11	0.265	0.825	1.054	1.005	0.061	0.09	0.930	0.893	0.132
Less hus-income	1.077	0.220	0.36	0.716	0.615	1.608	0.899	0.186	−0.51	0.607	0.600	1.347	0.99	0.204	−0.05	0.962	0.661	1.483

Notes: FCHPD, family care hours per day during the lockdown; Hus-FCHPD, husbands family care hours per day during the lockdown; working hours per week; Hus-education, education of husband; Hus-age, age of husband; Hus-income, annual income of husband; Less hus-income, whether the annual income of husband decreased during the lockdown.

**Table A3:** Ordinal logistic regression of worthless effect and general mental disorder with different factors in the total sample.

	Worthless						General Mental Disorder					
Characteristics	Odds Ratio	S.E.	*t*-Value	*p*-Value	[95% Conf. Interval]		Odds Ratio	S.E.	*t*-Value	*p*-Value	[95% Conf. Interval]	
FCHPD	1.002	0.061	0.03	0.975	0.889	1.129	0.934	0.054	−1.2	0.232	0.834	1.045
More family care	1.182	0.192	1.03	0.304	0.859	1.626	2.023	0.319	4.46	0.000	1.484	2.757
Job type	1.574	0.282	2.53	0.011	1.108	2.235	2.573	0.444	5.48	0.000	1.835	3.608
Annual income	1.185	0.072	2.78	0.005	1.051	1.335	1.149	0.065	2.47	0.014	1.029	1.283
Less income	1.363	0.29	1.46	0.145	0.899	2.067	1.601	0.326	2.31	0.021	1.074	2.387
Child	0.965	0.151	−0.23	0.820	0.71	1.312	1.028	0.151	0.19	0.849	0.771	1.371
Age	1.005	0.096	0.05	0.956	0.833	1.213	0.967	0.087	−0.37	0.713	0.811	1.154
Region	0.871	0.145	−0.83	0.406	0.629	1.206	1.067	0.165	0.42	0.674	0.788	1.446
Education	1.097	0.066	1.54	0.123	0.975	1.235	1.1	0.063	1.66	0.096	0.983	1.23
Hus-FCHPD	0.943	0.059	−0.95	0.345	0.834	1.066	0.987	0.058	−0.23	0.819	0.88	1.107
Hus-education	1.055	0.067	0.85	0.395	0.932	1.195	1.061	0.063	1.00	0.318	0.945	1.191
Hus-age	0.92	0.07	−1.10	0.271	0.794	1.067	0.908	0.065	−1.34	0.179	0.788	1.045
Hus-income	1.046	0.064	0.74	0.461	0.928	1.180	0.966	0.056	−0.6	0.956	0.863	1082
Less hus-income	0.975	0.196	−0.13	0.9000000000	0.657	1.447	0.897	0.174	−0.56	0.575	0.614	1.311

Notes: FCHPD, family care hours per day during the lockdown; Hus-FCHPD, husbands’ family care hours per day during the lockdown; working hours per week; Hus-education, education of husband; Hus-age, age of husband; Hus-income, annual income of husband; Less hus-income, whether the annual income of husband decreased during the lockdown.

## Appendix B. Questionnaire

Your age is			
A. 21–30 years old		B. 31–40 years old	
C. 41–50 years old		D. Above 50 years old	
Your region of registration			
A. Urban area		B. Rural area	
Your highest education level is			
A. High school or below	B. College	C. Bachelor	D. Master E. Doctor
The category of your job should belong to			
A. self-employed (employer)		B. employee	
Your annual income (RMB)			
A. 50,000 or below E. 200,000 above	B. 50,001–100,000	C. 100,001–150,000	D. 150,001–200,000
Has your income decreased during the lockdown period?			
No		Yes	
Do you have children?			
No		Yes	
Your family care hours per day during the period of lockdown			
A. 1 h or below E. 7 h above	B. 2–3 h	C. 4–5 h	D. 6–7 h
Compared to days before COVID-19, has your family care time during the lockdown increased?			
No		Yes	
Your husband’s age is			
A. 21–30 years	B. 31–40 years	C. 41–50 years	D. above 50 years
Your husband’s annual income is			
A. 50,000 or below E. 200,000 above.	B. 50,001–100,000	C. 100,001–150,000	D. 150,001–200,000
Your husband’s highest education level is			
A. High school or below	B. College	C. Bachelor	D. Master E. Doctor
The family care hours of your husband per day during the lockdown period			
A. 1 h or below E. 7 h above	B. 2–3 h	C. 4–5 h	D. 6–7 h
During the lockdown period, how often did you feel tired out for no good reason?			
A. None of the time E. All of the time	B. A little of the time	C. Some of the time	D. Most of the time
During the lockdown period, how often did you feel nervous?			
A. None of the time E. All of the time	B. A little of the time	C. Some of the time	D. Most of the time
During the lockdown period, how often did you feel too nervous to calm yourself down?			
A. None of the time E. All of the time	B. A little of the time	C. Some of the time	D. Most of the time
During the lockdown period, how often did you feel desperate?			
A. None of the time E. All of the time	B. A little of the time	C. Some of the time	D. Most of the time
During the lockdown period, how often did you feel restless?			
A. None of the time E. All of the time	B. A little of the time	C. Some of the time	D. Most of the time
During the lockdown period, how often did you feel too restless to stay still?			
A. None of the time E. All of the time	B. A little of the time	C. Some of the time	D. Most of the time
During the lockdown period, how often did you feel depressed?			
A. None of the time E. All of the time	B. A little of the time	C. Some of the time	D. Most of the time
During the lockdown period, how often did you feel that it is hard to finish anything?			
A. None of the time E. All of the time	B. A little of the time	C. Some of the time	D. Most of the time
During the lockdown period, how often did you feel so sad that nothing could cheer you up?			
A. None of the time E. All of the time	B. A little of the time	C. Some of the time	D. Most of the time
During the lockdown period, how often did you feel worthless?			
A. None of the time E. All of the time	B. A little of the time	C. Some of the time	D. Most of the time
